# Morphomania

**Published:** 1898-04-02

**Authors:** 


### MORPHOMANIA.

In an admirable clinical lecture delivered at Guy's Hospital, Dr. Hale White describes the symptoms and the consequences of what he calls "morphomania"—the craving for morphia. The first case mentioned is that of a lady, whom he found waiting for him one day on his arrival at home. He soon came to the conclusion that she was suffering from morphia, and, not being very ready to accept off-hand her promises that she would go straight home and throw away her syringe and her bottles, he went home with her. "This," he says, "is what I found in her room: Two four-ounce bottles of hypodermic injection of morphia. Why, gentlemen, that is almost enough to last the hospital a year! And then I found a whole stock of syringes; and, lastly, I came across the following prescription: ℞. Injectio morphinæ hypodermica ℥ii. To be used as directed. I think this is the most horrible prescription I ever heard of. I cannot understand any doctor doing such a thing. Look at the awful temptation she was exposed to. She had never had, and never could have, any children. She was in great trouble, was obliged to wander from health resort to health resort; she was not in a condition to be able to keep house properly; and, last of all, she suffered from chronic pain;—and he puts into her hands this prescription! I feel I shall have done well in this course of lectures if I have persuaded you never for any consideration, or under any circumstances whatever, to write such a prescription for any living person." The next case was that of a medical man, a case illustrating the moral degradation which so often accompanies the use of morphia; and the third that of a journalist who had wrecked himself by means of the same drug. In regard to the mental symptoms of morphomania, Dr. Hale White says that their essence is simply this—that its victims are liars, but that they lie with an object, that object being to get morphia. Again, a marked symptom is a gradual deterioration of method and punctuality, and a growing tendency to become slovenly, dirty, and untidy.

As to physical signs the patients are generally thin, sallow, and anæmic; they look prematurely old, their hair falls out, their teeth decay, their nails become brittle, and there is often great constipation. Then there is loss of sexual desire and of sexual power, and this occurs in women as well as in men. There may be amenorrhœa. They frequently have indigestion, the mouth is dry, and the pupils are often contracted. Slight pains, tremor, and ataxy, as shown, for example, in the writing, may occur; and occasionally the perspiration is increased, although as a rule the skin is dry.

The chief difficulty in the diagnosis arises from the patients being so deceitful, but this can be got over by isolating them for a day. There is such an irresistible craving that they confess at once.

As to prognosis, this is grave enough. Seventy per cent. relapse after being cured, and as practically no one gets well after taking to it a second time, out of the 70 per cent. of relapses there are no cures. In regard to treatment, Dr. Hale White advises the most absolute and complete isolation, after a thorough search for hidden morphia, and then a gradual reduction of the dose.

